# The Silent Threat: Unmasking Combined Post- and Pre-capillary Pulmonary Hypertension (CpcPH) in an Elderly Patient With Heart Failure With Preserved Ejection Fraction (HFpEF)

**DOI:** 10.7759/cureus.113174

**Published:** 2026-07-22

**Authors:** Prashant R Bhatt, Ashish Gupta, Anuva Ray, Ashutosh Sharma, Syed Raza, Thirumala Kammaripalle, Manoj Bhandari

**Affiliations:** 1 Internal Medicine, Cape Fear Valley Medical Center, Fayetteville, USA; 2 Cardiology, Cape Fear Valley Medical Center, Fayetteville, USA

**Keywords:** combined post capillary pulmonary hypertension, diagnostic delay, elderly patient, heart failure preserved ejection fraction, pulmonary hypertension, right heart catheterization, right heart failure, right ventricular dysfunction, syncope, vascular remodeling

## Abstract

Pulmonary hypertension (PH) is a progressive disorder associated with substantial morbidity and mortality, and its diagnosis may be delayed in older adults with multiple cardiopulmonary comorbidities because symptoms frequently overlap with more common cardiovascular and pulmonary conditions. Combined post- and pre-capillary pulmonary hypertension (CpcPH) is a severe phenotype characterized by pulmonary vascular remodeling superimposed on elevated left-sided filling pressures and is associated with worse outcomes than isolated post-capillary PH.

We report the case of a 77-year-old man with chronic hypoxic respiratory failure, heart failure with preserved ejection fraction (HFpEF), chronic atrial fibrillation, coronary artery disease, and obstructive sleep apnea who presented with progressive exertional dyspnea, syncope, and increasing oxygen requirements. His symptoms were initially attributed to HFpEF and coexisting cardiopulmonary conditions. Serial transthoracic echocardiography demonstrated progressive right ventricular enlargement, worsening right ventricular systolic function, interventricular septal flattening, and progressively increasing right ventricular systolic pressure (RVSP) from 46.6 to 87 mmHg. Right heart catheterization confirmed severe combined post- and pre-capillary pulmonary hypertension with a pulmonary capillary wedge pressure of 16 mmHg and pulmonary vascular resistance of 10.7 Wood units. Comprehensive evaluation excluded alternative causes of pre-capillary pulmonary hypertension. Because the severity of pulmonary vascular disease appeared disproportionate to isolated HFpEF-related pulmonary hypertension, the patient underwent multidisciplinary evaluation at a specialized pulmonary hypertension center, where an individualized treatment strategy with intravenous epoprostenol and sildenafil was initiated, resulting in improvement in functional status and oxygen requirements during hospitalization.

This case highlights the diagnostic challenges of CpcPH in patients with HFpEF and multiple cardiopulmonary comorbidities. Progressive right ventricular remodeling, syncope, and worsening oxygen requirements should prompt consideration of pulmonary hypertension when clinical deterioration appears disproportionate to the underlying cardiopulmonary disease. Early recognition, serial echocardiographic assessment, timely right heart catheterization, and referral to specialized pulmonary hypertension centers are essential for establishing an accurate diagnosis and guiding individualized management.

## Introduction

Pulmonary hypertension (PH) is a progressive hemodynamic disorder characterized by elevated pulmonary arterial pressure that increases right ventricular (RV) afterload and, if left unrecognized or untreated, may progress to RV dysfunction, right heart failure, and premature death [1]. According to the 2022 European Society of Cardiology (ESC)/European Respiratory Society (ERS) guidelines, PH is defined as a mean pulmonary arterial pressure (mPAP) >20 mmHg at rest measured by right heart catheterization (RHC), the gold standard for diagnosis [1].

Pulmonary hypertension is classified into five clinical groups based on the underlying pathophysiology: Group 1, pulmonary arterial hypertension (PAH); Group 2, PH due to left heart disease; Group 3, PH associated with lung disease and/or hypoxia; Group 4, PH associated with pulmonary artery obstruction, including chronic thromboembolic pulmonary hypertension; and Group 5, PH with unclear or multifactorial mechanisms [1]. This classification is clinically important because the underlying pathophysiology, diagnostic evaluation, and treatment strategies differ substantially among the groups.

Group 2 PH is the most common form of PH and frequently complicates heart failure with preserved ejection fraction (HFpEF) [1-3]. Hemodynamically, post-capillary PH is defined by an mPAP >20 mmHg and a pulmonary arterial wedge pressure (PAWP) >15 mmHg. Based on pulmonary vascular resistance (PVR), it is further classified as isolated post-capillary PH (PVR ≤2 Wood units) or combined post- and pre-capillary pulmonary hypertension (CpcPH; PVR >2 Wood units) [1]. CpcPH represents a more advanced disease phenotype characterized by pulmonary vascular remodeling superimposed on elevated left-sided filling pressures and is associated with more severe RV dysfunction, reduced exercise capacity, increased hospitalization rates, and poorer survival than isolated post-capillary PH [4,5].

Distinguishing Group 1 PAH from Group 2 PH is essential because management differs substantially. PAH-specific therapies, including prostacyclin pathway agents, endothelin receptor antagonists, phosphodiesterase-5 inhibitors, and soluble guanylate cyclase stimulators, are established treatments for appropriately selected patients with Group 1 PAH [1]. In contrast, these therapies are not routinely recommended for patients with Group 2 PH because randomized clinical trials have not demonstrated consistent clinical benefit and may increase the risk of pulmonary edema in the setting of elevated left-sided filling pressures [1]. Consequently, management of Group 2 PH focuses on optimizing the underlying cardiac disease and guideline-directed heart failure therapy, while patients with severe PH, a significant pre-capillary component, or diagnostic uncertainty should be referred to specialized pulmonary hypertension centers for comprehensive evaluation [1,6].

The diagnosis of CpcPH is often challenging because its clinical manifestations overlap with those of common cardiopulmonary disorders. Symptoms such as exertional dyspnea, fatigue, peripheral edema, and syncope are frequently attributed to HFpEF, atrial fibrillation, coronary artery disease, obstructive sleep apnea, or other age-related comorbidities rather than progressive pulmonary vascular disease [1-3]. Consequently, recognition is often delayed until the disease has reached an advanced stage.

Syncope is a high-risk feature of advanced PH and often reflects impaired RV function with an inability to augment cardiac output during exertion [1]. In older adults with multiple comorbidities, however, recurrent syncope is frequently attributed to orthostatic hypotension, medication effects, or cardiac arrhythmias, further delaying recognition of PH [7].

We present a case of severe combined post- and pre-capillary pulmonary hypertension in an elderly patient with recurrent syncope whose diagnosis was delayed by overlapping cardiopulmonary comorbidities and initially nonspecific clinical findings, highlighting the diagnostic challenges and therapeutic complexity of advanced CpcPH.

## Case presentation

A 77-year-old man with chronic hypoxic respiratory failure requiring 2 L/min of home oxygen, hypertension, hyperlipidemia, hypothyroidism, obstructive sleep apnea, chronic atrial fibrillation, and coronary artery disease status post coronary artery bypass grafting approximately 8 years earlier presented with progressive exertional dyspnea and worsening bilateral lower-extremity edema. His oxygen saturation was 92% on his baseline oxygen requirement, heart rate was 94 beats/min, and blood pressure was 132/90 mmHg. Initial laboratory evaluation, including a complete blood count and comprehensive metabolic panel, was unremarkable. Brain natriuretic peptide (BNP) was elevated at 960 pg/mL compared with his baseline of 200-300 pg/mL, while chest radiography showed no acute cardiopulmonary abnormality. Following treatment with intravenous diuretic therapy, optimization of inhaled bronchodilator therapy, supplemental oxygen, and management of a suspected chronic obstructive pulmonary disease exacerbation, the patient's respiratory symptoms and volume status improved. After clinical stabilization, he preferred outpatient evaluation and was discharged with plans for transthoracic echocardiography (TTE) and close follow-up.

**Figure 1:**
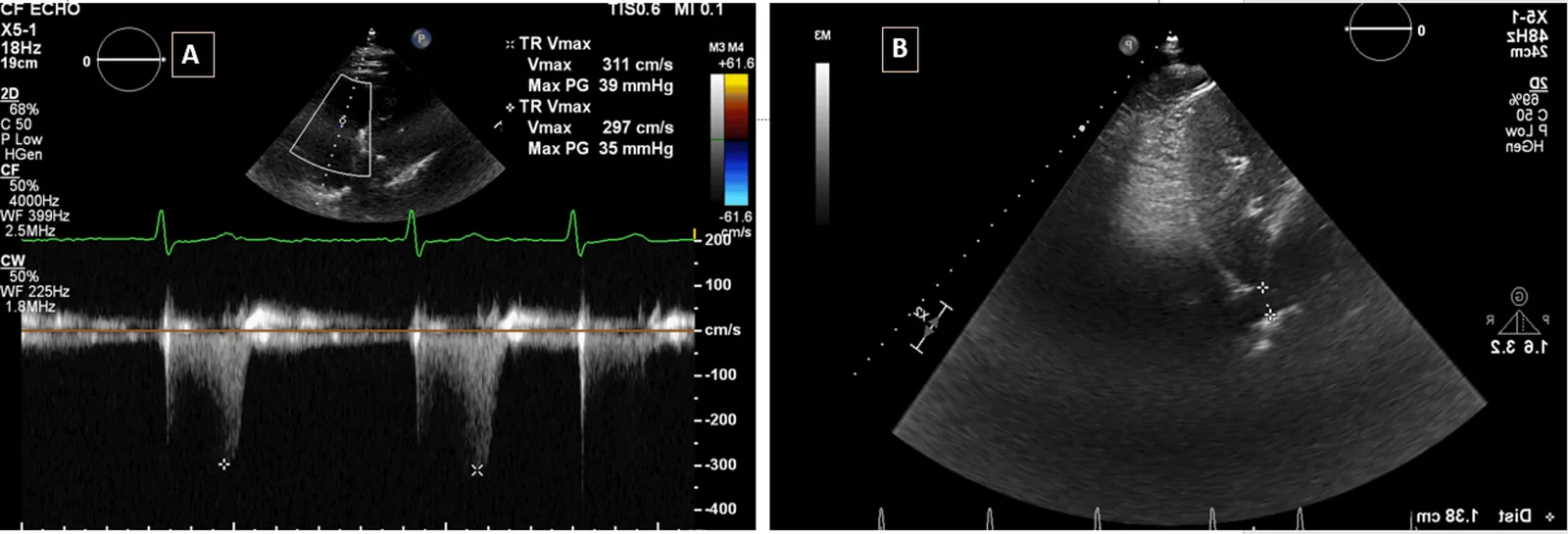
Echocardiographic estimation of mildly elevated right ventricular systolic pressure at initial presentation (A) Continuous-wave Doppler of the tricuspid regurgitation jet used for estimation of right ventricular systolic pressure (RVSP). The formal echocardiographic interpretation reported a peak tricuspid regurgitation velocity of 3.02 m/s with an assumed right atrial pressure of 10 mmHg, yielding an estimated right ventricular systolic pressure of 46.6 mmHg. (B) Subcostal view demonstrating the inferior vena cava (diameter 1.38 cm), which was used in conjunction with respiratory collapsibility for estimation of right atrial pressure.

Three months later, he was readmitted with worsening dyspnea and volume overload. TTE demonstrated a preserved left ventricular ejection fraction (>55%) with pseudonormal diastolic filling, consistent with heart failure with preserved ejection fraction (HFpEF), and a mildly elevated right ventricular systolic pressure (RVSP) of 46.6 mmHg (Figure 1). BNP had increased to 1,028 pg/mL. Given the preserved systolic function, diastolic dysfunction, and only mildly elevated pulmonary pressures, his presentation was attributed to HFpEF-related congestion, and diuretic therapy together with guideline-directed medical therapy (GDMT) was optimized accordingly [6].

Over the following year, he developed progressive exercise intolerance and recurrent syncopal episodes, including one during ambulation. Given his chronic hypoxic respiratory failure, HFpEF, atrial fibrillation, and multiple cardiovascular comorbidities, these episodes were initially attributed to orthostatic hypotension, worsening hypoxemia, medication effects, or intermittent rapid ventricular response. Follow-up TTE continued to demonstrate preserved left ventricular systolic function with mild right atrial enlargement, while ambulatory cardiac monitoring confirmed chronic atrial fibrillation without additional arrhythmias.

**Figure 2:**
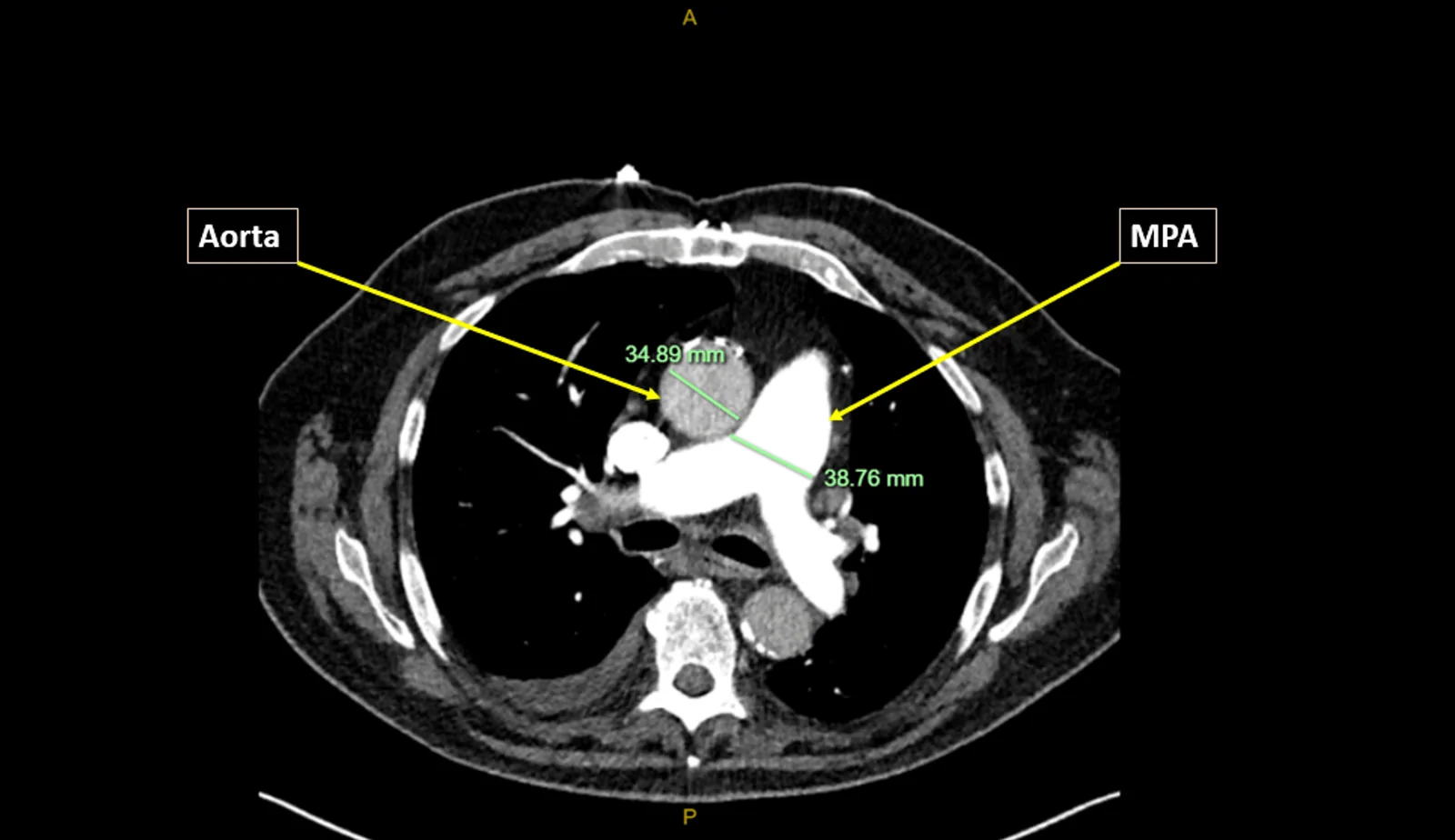
Contrast-enhanced CT chest demonstrating main pulmonary artery dilatation Axial contrast-enhanced CT pulmonary angiography demonstrating main pulmonary artery (MPA) dilatation measuring 38.76 mm, exceeding the diameter of the ascending aorta (Ao; 34.89 mm), consistent with pulmonary hypertension.

**Figure 3:**
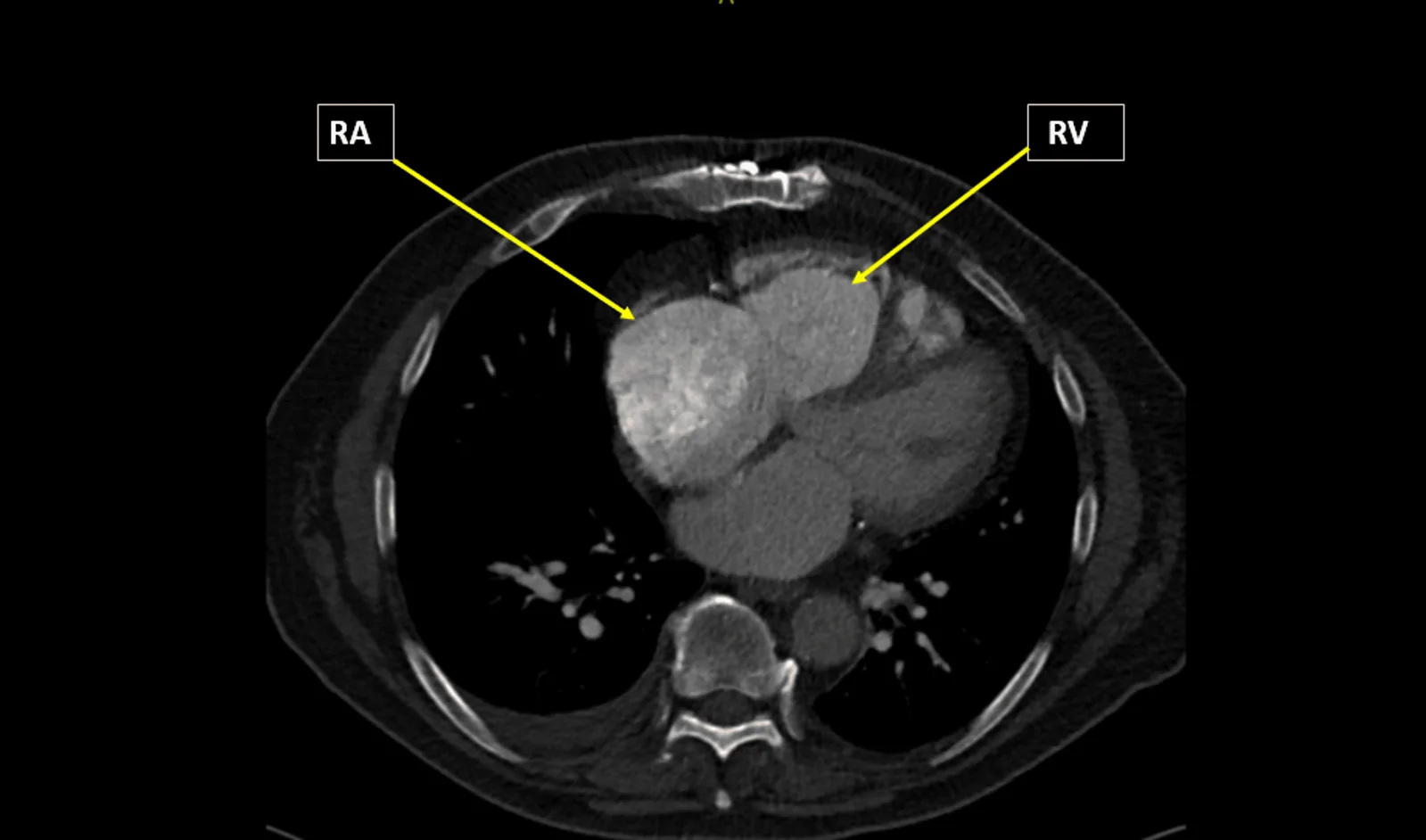
Contrast-enhanced CT chest demonstrating right-sided cardiac chamber enlargement Axial contrast-enhanced CT demonstrating right atrial (RA) and right ventricular (RV) enlargement, consistent with chronic right ventricular pressure overload secondary to pulmonary hypertension.

Two years after his initial presentation, he returned with recurrent syncope, worsening functional capacity, and increasing oxygen requirements beyond his baseline of 2 L/min. Computed tomography angiography (CTA) of the chest demonstrated cardiomegaly, right-sided chamber enlargement, a right pleural effusion, and dilation of the main pulmonary artery, findings suggestive of pulmonary hypertension (Figures 2, 3).

**Figure 4:**
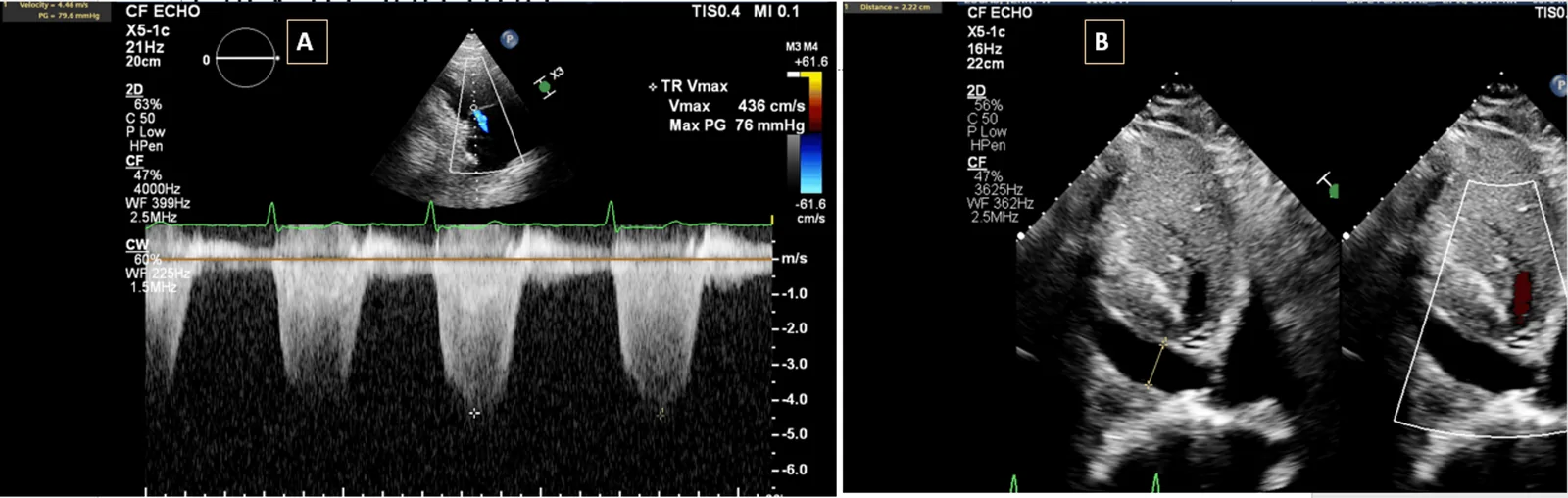
Follow-up echocardiographic assessment of right ventricular systolic pressure (A) Continuous-wave Doppler of the tricuspid regurgitation jet used for estimation of right ventricular systolic pressure (RVSP). The formal transthoracic echocardiographic interpretation reported a peak tricuspid regurgitation velocity of 4.39 m/s. (B) Subcostal view demonstrating a dilated inferior vena cava (IVC) measuring 2.22 cm. Based on IVC size and respiratory collapsibility, the formal echocardiographic interpretation assumed a right atrial pressure of 10 mmHg, yielding an estimated RVSP of 87.1 mmHg, consistent with severe pulmonary hypertension.

**Figure 5:**
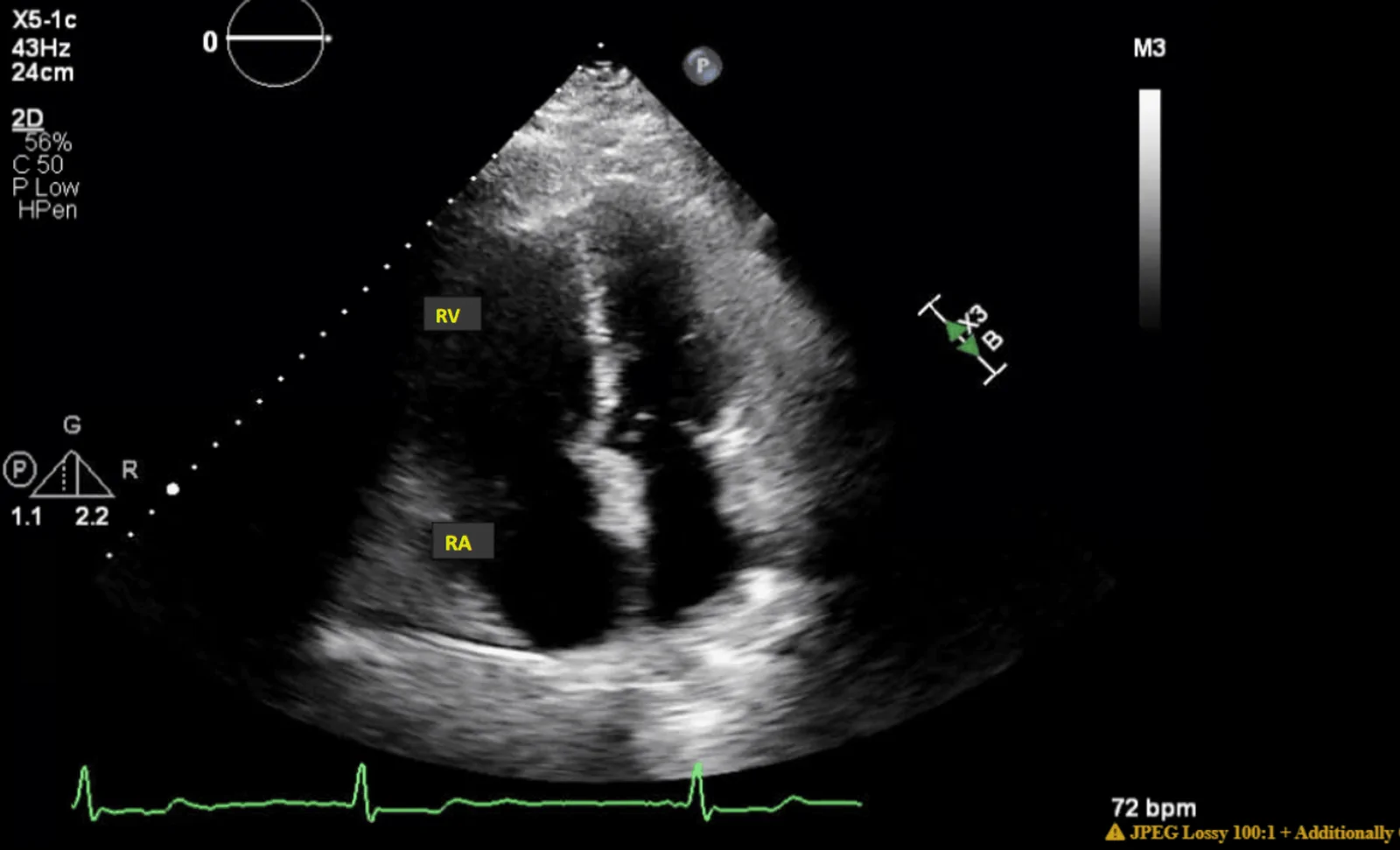
Apical four-chamber echocardiographic view demonstrating right heart chamber enlargement Apical four-chamber view demonstrating marked enlargement of the right ventricle (RV) and right atrium (RA), consistent with right heart chamber dilatation.

**Figure 6:**
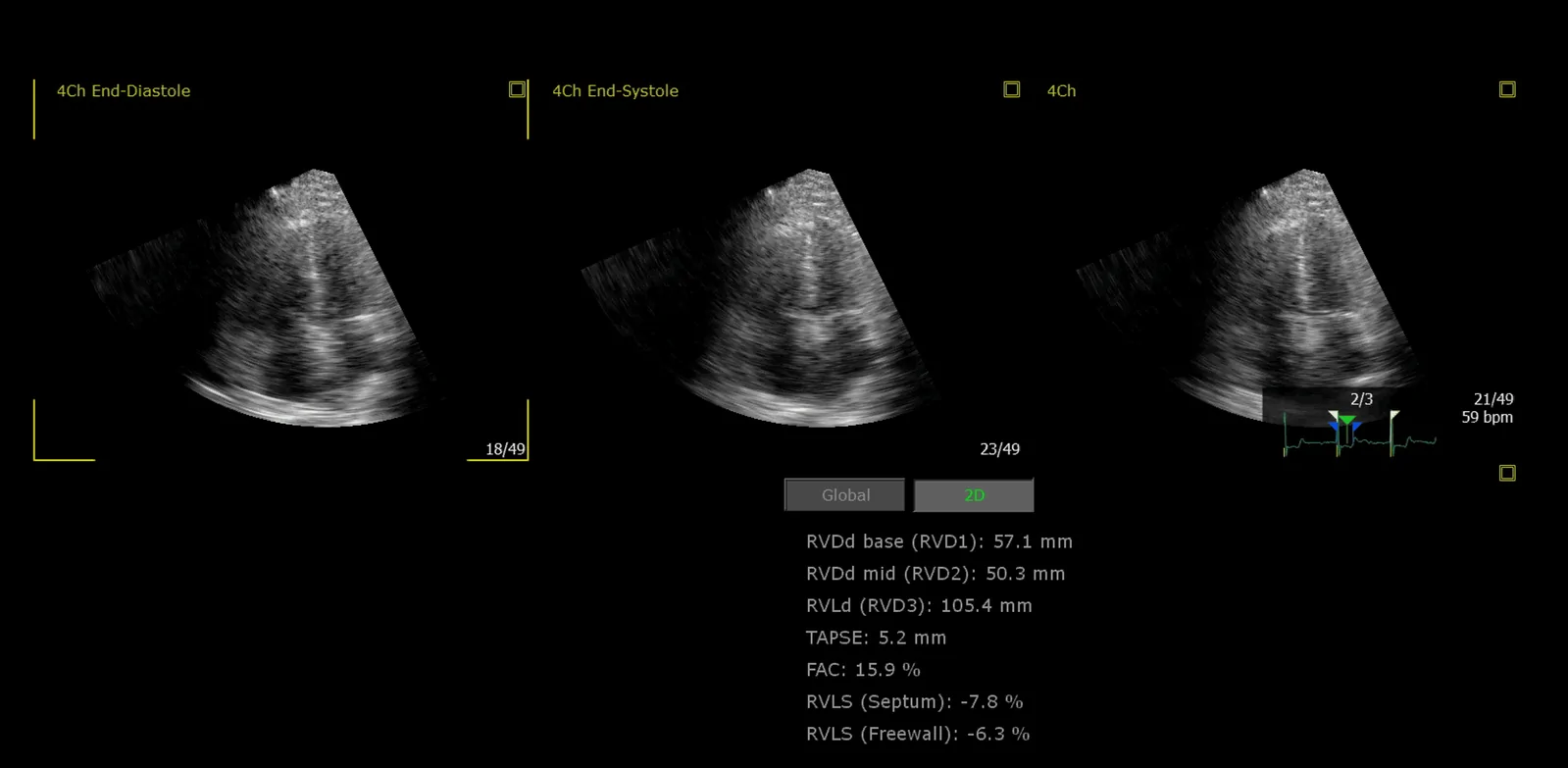
Quantitative echocardiographic assessment of right ventricular size and systolic function Quantitative two-dimensional echocardiographic assessment demonstrating severe right ventricular enlargement, with a basal diameter (RVD1) of 57.1 mm, mid-cavity diameter (RVD2) of 50.3 mm, and longitudinal diameter (RVD3) of 105.4 mm. Right ventricular systolic function was severely impaired, with a tricuspid annular plane systolic excursion (TAPSE) of 5.2 mm and fractional area change (FAC) of 15.9%.

**Figure 7:**
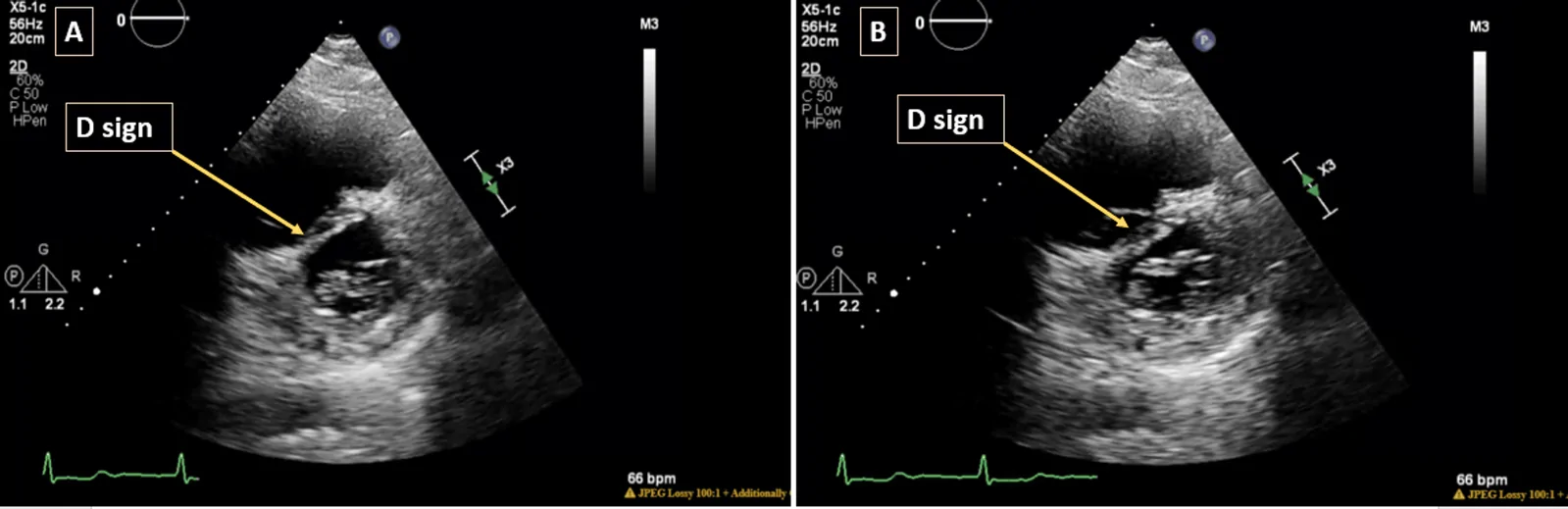
Parasternal short-axis views demonstrating persistent interventricular septal flattening consistent with right ventricular pressure overload (A) End-systolic parasternal short-axis view demonstrating a D-shaped left ventricle due to interventricular septal flattening; (B) End-diastolic parasternal short-axis view demonstrating persistent interventricular septal flattening with a D-shaped left ventricle

Follow-up TTE during this admission revealed marked progression, demonstrating mild-to-moderate right ventricular enlargement, reduced right ventricular systolic function, interventricular septal flattening, and a severely elevated RVSP of 87 mmHg (Figures 4-7). Compared with earlier studies, the extent of right ventricular remodeling and pulmonary hypertension exceeded what would typically be expected in uncomplicated HFpEF, prompting referral to a tertiary care center with a pulmonary hypertension center for further evaluation.

Right and left heart catheterization confirmed severe combined post- and pre-capillary pulmonary hypertension. Hemodynamic findings are summarized in Table 1.

**Table 1:** Hemodynamic measurements from heart catheterization mPAP: mean pulmonary arterial pressure; PCWP: pulmonary capillary wedge pressure; PVR: pulmonary vascular resistance; RAP: right atrial pressure; RV: right ventricle; TPG: transpulmonary gradient; DPG: diastolic pressure gradient

Parameter	Value	Normal Range
Mean pulmonary arterial pressure (mPAP)	61 mmHg	8-20 mmHg
Pulmonary capillary wedge pressure (PCWP)	16 mmHg	≤15 mmHg
Pulmonary vascular resistance (PVR)	10.7 Wood units	0.3-2.0 WU
Right atrial pressure (RAP)	15 mmHg	2-6 mmHg
Right ventricular pressure	109/4/15 mmHg	15-30/0-8/0-8 mm Hg
Cardiac output (Fick)	4.2 L/min	4.0-8.0 L/min
Cardiac index	1.9 L/min/m²	2.5-4.0 L/min/m²
Transpulmonary gradient (TPG)	45 mmHg	<12 mmHg
Diastolic pressure gradient (DPG)	23 mmHg	1-3 mmHg

Moreover, an extensive evaluation, including ventilation-perfusion (V/Q) scanning, chest computed tomography demonstrating no significant parenchymal lung disease, HIV testing, autoimmune serologies, serum protein electrophoresis, and serum viscosity studies, excluded chronic thromboembolic pulmonary hypertension, connective tissue disease-associated pulmonary hypertension, and other secondary causes.

The patient subsequently underwent multidisciplinary evaluation by cardiology and pulmonology specialists. Although the hemodynamic profile fulfilled criteria for combined post- and pre-capillary pulmonary hypertension, the severity of pulmonary vascular involvement was considered disproportionate to isolated HFpEF-related (World Health Organization (WHO) Group 2) pulmonary hypertension, precluding definitive classification into a single WHO PH group. Given his recurrent syncope, progressive functional decline, increasing oxygen requirements, markedly reduced cardiac index, and WHO functional class IV symptoms, an individualized treatment strategy with continuous intravenous epoprostenol and sildenafil was initiated at the specialized pulmonary hypertension center [1].

Intravenous epoprostenol was gradually uptitrated under close inpatient monitoring. The patient's exercise tolerance, oxygen requirements, and volume status improved during hospitalization. Metoprolol was carefully titrated down to optimize hemodynamics while maintaining adequate ventricular rate control in chronic atrial fibrillation. A tunneled Hickman catheter was placed for long-term epoprostenol infusion, and sildenafil was initiated with plans to transition to tadalafil in the outpatient setting. After completing infusion education, he was discharged home on continuous intravenous epoprostenol, sildenafil, optimized diuretic therapy, and close follow-up at the specialized pulmonary hypertension center.

## Discussion

pH remains a diagnostic challenge in older adults with multiple cardiopulmonary comorbidities because its clinical manifestations frequently overlap with those of more common cardiovascular and pulmonary disorders. Consequently, progressive pulmonary vascular disease may remain unrecognized until advanced right ventricular (RV) dysfunction and significant hemodynamic compromise develop [1]. This challenge is particularly evident in patients with HFpEF, in whom exertional dyspnea, exercise intolerance, peripheral edema, and increasing oxygen requirements are often attributed to worsening left-sided heart failure rather than progression of pulmonary vascular disease [2,3].

PH due to left heart disease is the most common form of PH and frequently complicates HFpEF [1]. Although many patients have isolated post-capillary PH, approximately 20-30% develop CpcPH, reflecting pulmonary vascular remodeling beyond passive transmission of elevated left-sided filling pressures [4,8]. Compared with isolated post-capillary PH, CpcPH is associated with higher pulmonary vascular resistance, more pronounced RV dysfunction, reduced exercise capacity, increased heart failure hospitalizations, and worse survival [4,5,8]. Recognizing this transition is clinically important because it identifies patients with a more advanced disease phenotype and poorer prognosis.

Our patient's clinical course illustrates this progression. Initial findings, including preserved left ventricular systolic function, elevated natriuretic peptide levels, volume overload, and echocardiographic evidence of diastolic dysfunction, were consistent with HFpEF-related congestion. However, serial echocardiography demonstrated progressive RV enlargement, declining RV systolic function, and steadily increasing pulmonary pressures. These findings became increasingly disproportionate to uncomplicated HFpEF, prompting invasive hemodynamic assessment, which confirmed severe CpcPH.

Recurrent syncope was an important marker of disease progression rather than an isolated clinical event. Syncope is a well-recognized, high-risk feature of advanced PH and reflects impaired RV adaptation with an inability to augment cardiac output during periods of increased demand [1,5,9]. In this patient, however, chronic atrial fibrillation, chronic hypoxic respiratory failure, and suspected orthostatic hypotension provided several plausible alternative explanations, delaying recognition of progressive pulmonary vascular disease. This case highlights the importance of considering PH in patients with recurrent syncope accompanied by otherwise unexplained clinical deterioration, particularly when serial imaging demonstrates progressive right-sided cardiac abnormalities [1,9].

Serial echocardiography played a central role in identifying disease progression by demonstrating worsening RV enlargement, increasing RV systolic pressure, declining RV systolic function, and interventricular septal flattening, all recognized markers of advanced PH and adverse prognosis [1,5]. Nevertheless, echocardiography cannot reliably distinguish isolated post-capillary PH from CpcPH or accurately define the severity of pulmonary vascular remodeling. Right heart catheterization therefore remains the gold standard for establishing the hemodynamic phenotype, assessing disease severity, and guiding management [1].

Right heart catheterization confirmed severe CpcPH, demonstrating markedly elevated mean pulmonary arterial pressure, pulmonary capillary wedge pressure, pulmonary vascular resistance, transpulmonary gradient, and diastolic pressure gradient, together with elevated right atrial pressure and a markedly reduced cardiac index. These findings confirmed advanced pulmonary vascular remodeling superimposed on elevated left-sided filling pressures rather than passive pulmonary venous hypertension alone [1,4]. The markedly elevated pulmonary vascular resistance (10.7 Wood units) and reduced cardiac index provided a hemodynamic explanation for the patient's progressive functional decline, worsening exercise intolerance, and recurrent syncope [5,9].

Given the severity of these hemodynamic abnormalities, a comprehensive evaluation for alternative causes of pre-capillary PH was appropriate. Chronic thromboembolic disease, significant parenchymal lung disease, connective tissue disease, HIV infection, paraproteinemia, and hyperviscosity syndromes were excluded through multimodality imaging and laboratory investigations. Although the hemodynamic profile fulfilled diagnostic criteria for CpcPH, the severity of pulmonary vascular disease was considered disproportionate to isolated HFpEF-related (WHO Group 2) PH, preventing definitive classification into a single WHO PH group despite an extensive diagnostic evaluation [1].

Given the patient's advanced clinical presentation, severe hemodynamic impairment, and pulmonary vascular disease disproportionate to isolated HFpEF-related pulmonary hypertension, management was individualized following multidisciplinary evaluation at a specialized pulmonary hypertension center. An individualized treatment strategy with continuous intravenous epoprostenol and sildenafil was therefore initiated under close inpatient monitoring.

Importantly, this treatment strategy should not be interpreted as standard therapy for isolated WHO Group 2 PH or CpcPH. Current guidelines do not recommend the routine use of pulmonary arterial hypertension-targeted therapies in PH due to left heart disease because randomized clinical trials have not demonstrated consistent clinical benefit and have raised concerns regarding adverse outcomes, including an increased risk of pulmonary edema in patients with elevated left-sided filling pressures [1,10]. Instead, management should focus on optimizing the underlying cardiac disease. In contrast, patients with severe or disproportionate pulmonary vascular disease, diagnostic uncertainty, or progressive RV failure despite guideline-directed therapy should be referred to specialized pulmonary hypertension centers for comprehensive multidisciplinary evaluation [1,10].

This case highlights how progressive pulmonary vascular disease may remain concealed in patients with HFpEF and multiple overlapping cardiopulmonary comorbidities. The combination of recurrent syncope, progressive right ventricular remodeling, worsening functional capacity, and severe hemodynamic abnormalities should prompt clinicians to reconsider the diagnosis rather than attributing these findings exclusively to progression of left-sided heart disease [1,4,5]. Such patients benefit from comprehensive evaluation at specialized pulmonary hypertension centers, where multidisciplinary expertise can facilitate accurate phenotyping and individualized management [1,10].

## Conclusions

This case highlights the diagnostic challenges of CpcPH in an elderly patient with HFpEF and multiple cardiopulmonary comorbidities. Coexisting cardiopulmonary conditions can obscure the recognition of progressive pulmonary vascular disease, delaying diagnosis until advanced right ventricular dysfunction and hemodynamic compromise develop. Clinicians should maintain a high index of suspicion for pulmonary hypertension when symptoms, syncope, or progressive right-sided cardiac abnormalities appear disproportionate to the underlying cardiopulmonary disease. Early recognition, serial echocardiographic assessment, timely right heart catheterization, and referral to specialized pulmonary hypertension centers are essential for establishing an accurate diagnosis and guiding individualized management in patients with CpcPH. This case underscores the importance of a multidisciplinary approach in evaluating complex pulmonary hypertension to ensure accurate phenotyping and individualized management.
